# Basophil Reactivity as Biomarker in Immediate Drug Hypersensitivity Reactions—Potential and Limitations

**DOI:** 10.3389/fphar.2016.00171

**Published:** 2016-06-17

**Authors:** Markus Steiner, Andrea Harrer, Martin Himly

**Affiliations:** ^1^Division Allergy and Immunology, Department Molecular Biology, University of SalzburgSalzburg, Austria; ^2^Laboratory for Immunological and Molecular Cancer Research, Paracelsus Medical UniversitySalzburg, Austria; ^3^Department Neurology, Paracelsus Medical UniversitySalzburg, Austria

**Keywords:** antibiotics, basophil activation test, biologicals, chemotherapeutics, fluoroquinolones, NMBAs, NSAIDs, RCM

## Abstract

Immediate drug hypersensitivity reactions (DHRs) resemble typical immunoglobulin E (IgE)-mediated symptoms. Clinical manifestations range from local skin reactions, gastrointestinal and/or respiratory symptoms to severe systemic involvement with potential fatal outcome. Depending on the substance group of the eliciting drug the correct diagnosis is a major challenge. Skin testing and *in vitro* diagnostics are often unreliable and not reproducible. The involvement of drug-specific IgE is questionable in many cases. The culprit substance (parent drug or metabolite) and potential cross-reacting compounds are difficult to identify, patient history and drug provocation testing often remain the only means for diagnosis. Hence, several groups proposed basophil activation test (BAT) for the diagnosis of immediate DHRs as basophils are well-known effector cells in allergic reactions. However, the usefulness of BAT in immediate DHRs is highly variable and dependent on the drug itself plus its capacity to spontaneously conjugate to serum proteins. Stimulation with pure solutions of the parent drug or metabolites thereof vs. drug-protein conjugates may influence sensitivity and specificity of the test. We thus, reviewed the available literature about the use of BAT for diagnosing immediate DHRs against drug classes such as antibiotics, radio contrast media, neuromuscular blocking agents, non-steroidal anti-inflammatory drugs, and biologicals. Influencing factors like the selection of stimulants or of the identification and activation markers, the stimulation protocol, gating strategies, and cut-off definition are addressed in this overview on BAT performance. The overall aim is to evaluate the suitability of BAT as biomarker for the diagnosis of immediate drug-induced hypersensitivity reactions.

## Key players in the immediate-type allergic effector phase

**Figure 1:**
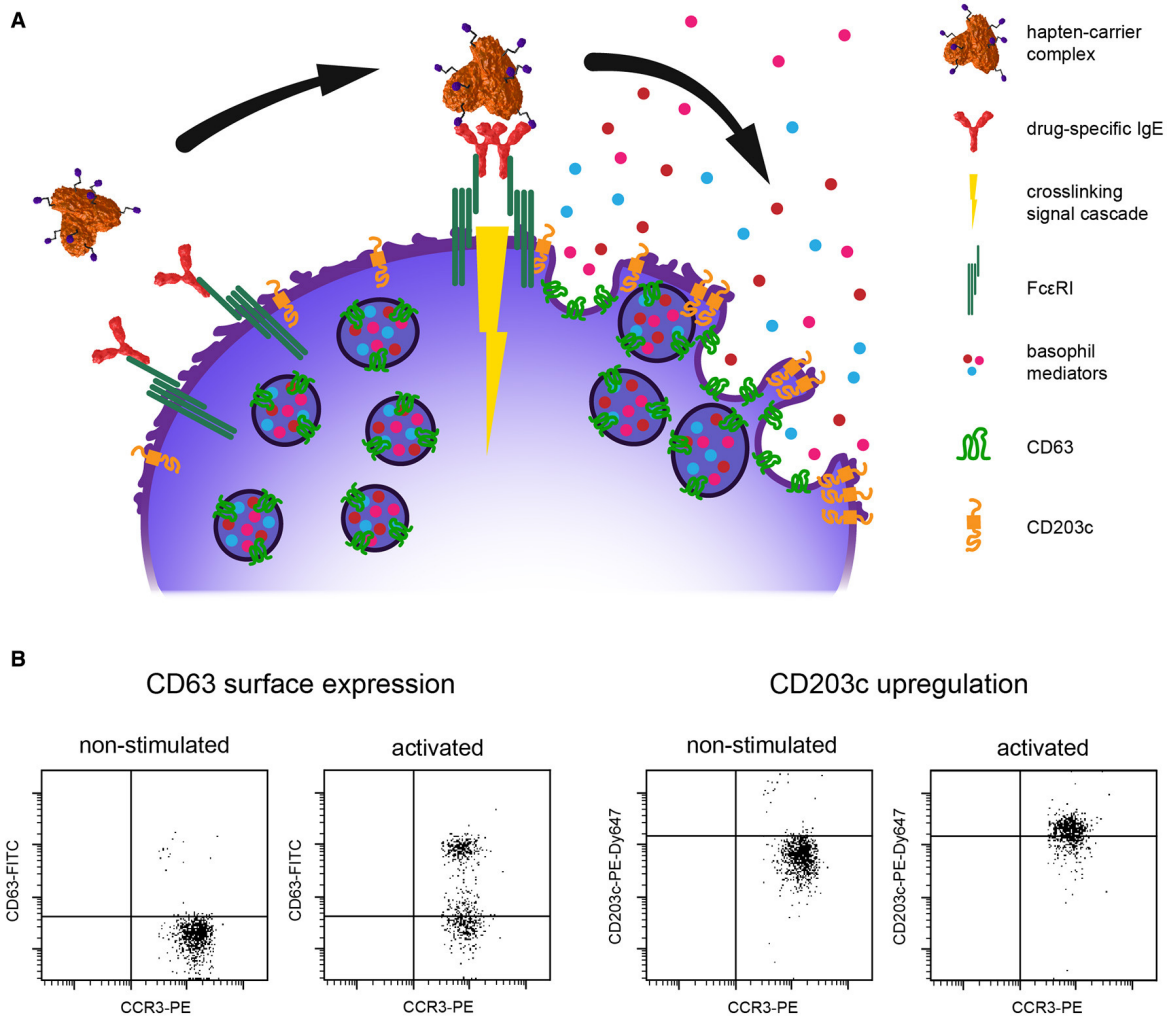
**(A)** Schematic illustration of IgE-mediated cross-linking. Upon binding of the hapten-carrier complex to the drug-specific IgE captured on high affinity IgE receptor FcεRI, basophils react by degranulation and mediator release. CD63 is transferred from the vesicle to the plasma membrane and CD203c is upregulated, thus both serve as activation markers. **(B)** Flow cytometry dot plots of typical activation patterns for CD63 and CD203c; CCR3, basophil identification marker; PE, phycoerthrin label; FITC, fluorescein label; PE-Dy647, tandem label.

Key characteristic of allergic effector cells in immediate-type allergy is allergen-specific IgE bound to the high affinity IgE receptor, i.e., FcεRI, on the cell surface. Capturing of allergens by surface IgE results in FcεRI crosslinking and elicits the acute phase of the allergic response involving the sudden release of vasoactive mediators into the tissue and/or circulation. This sudden activation process and mediator release is termed anaphylactic degranulation (depicted in Figure 1A) and may induce life-threatening anaphylaxis (Hoffmann, 2015). Mast cells and basophils both share these key characteristics. Mast cells reside in the tissue and are considered primary allergic effector cells. Basophils are peripheral blood granulocytes, easily accessible via venipuncture and a well-established surrogate for allergy diagnosis (MacGlashan, 2013).

## The basophil activation test

Basophil activation can be measured by flow cytometry and multicolor staining with fluorescent-labeled detection antibodies targeting specific identification and activation markers on the surface of basophils. The most common identification strategies use surface IgE, eotaxin CC chemokine receptor 3 (CCR3), the combination of interleukin 3 receptor alpha chain CD123^high^ with human leukocyte antigen HLA-DR^neg^, the combination of prostaglandin D_2_ receptor CRTH-2^high^ with CD3^neg^, or the basophil-specific ectonuclease CD203c. Table 1 provides an overview of basophil identification strategies in drug hypersensitivity research which we numbered “strategy 1” through “strategy 6” according to the frequency they have been used. For basophil activation the degranulation marker lysosomal-associated membrane glycoprotein-3 (LAMP-3), also termed CD63, or upregulation of CD203c are determined. Thus, changes in activation state can be quantified on a single cell basis. Degranulation means fusion of specific intracellular vesicles filled with preformed mediators, the so-called granules, with the plasma membrane and a transition of CD63 from inside out. The result is a sudden and pronounced rise, i.e., log-shift, of the fluorescence intensity signal, in the detection of surface CD63. Concomitantly, upregulation of CD203c has been observed which can be detected as significant increase in the mean fluorescence intensity signal of the CD203c detection antibody. Flow cytometry dot plots for both activation scenarios are depicted in Figure 1B. For further details about assay parameters of BAT the interested reader is referred to the comprehensive review of McGowan and Saini (2013).

**Table 1:** **Overview on BAT applications for different drug classes, identification markers, acquisition conditions, observed activation patterns, and verification of IgE**.

**References**	**Drugs**	**Drug-specific serum IgE**	**Anti-coagulant**	**Basophil activation test protocols**				
				**Basophil identification strategies**	**Stimulation conditions**	**Acquisition details**		
					**Time [min] at 37°C**	**Activation marker**	**Basophils (n)**	**Cut-off**
**1. BETA-LACTAM ANTIBIOTICS**								
Eberlein et al., 2010	PCN G, PCN V, PPL, MDM, AMP, AX, CFX	Detected	EDTA	Strategy 1: SSC^low^, IgE^pos^	15	CD63	300	≥5%, SI ≥ 2
Abuaf et al., 2008	AX, AMP, CFX,	Not detected	HEP	Strategy 4^+^: SSC^low^, CD33^dim^, CD45^pos^, IgE^pos^	30	CD63, CD203c	200	Mean n.c.+ 2 × *SD*
De Week et al., 2009	PPL, MDM, BPN, AX, AMP, CEF	Detected	EDTA	Strategy 1: SSC^low^, IgE^pos^	40	CD63	500	≥5%, SI ≥ 2
Garcia-Ortega and Marin, 2010	AX	Detected	HEP	Strategy 1: SSC^low^, IgE^pos^	10^*^ + 20	CD63	1000	≥5%, SI ≥ 2
Sanz et al., 2002	BP, AMP, AX, MDM, PPL	Detected	ACD	Strategy 1: SSC^low^, IgE^pos^	40	CD63	500	≥5%, SI ≥ 2
Torres et al., 2010a	AX, DKP	Detected	HEP	Strategy 1: SSC^low^, IgE^pos^	10^*^ + 20	CD63	1000	SI(%)>2
Torres et al., 2004	AX, BP, BPP, AMP, MDM	Detected	HEP	Strategy 1: SSC^low^, IgE^pos^	10^*^ + 20	CD63	1000	SI(%)>2
Torres et al., 2011	AX	Detected	HEP	Strategy 1: SSC^low^, IgE^pos^	10^*^ + 20	CD63	1000	SI(%)>2
**2. FLUOROQUINOLONES**								
Aranda et al., 2011	CPX, LVX, MOX	Detected	HEP	Strategy 1: SSC^low^, IgE^pos^	30	CD63	250–500	≥5%, SI ≥ 2
Mayorga et al., 2013	CPX, MOX	Not detected	HEP	Strategy 1: SSC^low^, IgE^pos^	30	CD63	250–500	≥5%, SI ≥ 2
Lobera et al., 2010	CPX, LVX, MOX, NFX	Not detected	HEP	Strategy 2: SSC^low^, CD123^pos^, HLA-DR^neg^	15	CD63	200	≥5%, SI ≥ 2
Seitz et al., 2009	CPX, OFX, LVX, MOX	Not detected	EDTA	Strategy 1: SSC^low^, IgE^pos^	n.a.	CD63	n.a.	n.a.
Rouzaire et al., 2012	LVX, OFX, CPX, MOX, LMX, NFX, FLU, PPA	Not detected	HEP	Strategy 1^+^: SSC^low^, IgE^pos^, CD45^pos^	10	CD203c	n.a.	>10%
Blanca-Lopez et al., 2013	NFX, CPX, LVX, MOX	n.a	HEP	Strategy 1: SSC^low^, IgE^pos^	30	CD63	250–500	≥5%, SI ≥ 2
Ben Said et al., 2010b	CPX, NFX, OFX	Not detected	n.a.	n.a.	n.a.	CD203c	n.a.	n.a.
**3. NEURO-MUSCULAR BLOCKING AGENTS (NMBAs)**								
Ebo et al., 2007	ROC, SUX, MSO4, PHO	Detected	HEP	n.a.	n.a.	n.a.	n.a.	n.a.
Leysen et al., 2013	ROC, VEC, ATR, cATR, SUX	Detected	HEP	Strategy 1: SSC^low^, IgE^pos^	20	CD63, CD203c	n.a.	n.a.
Sainte-Laudy et al., 2006	ROC, VEC, cATR, SUX	Detected	EDTA	Strategy 1: SSC^low^, IgE^pos^	30	CD63	500	SI > 2
Ebo et al., 2006	ROC	Not detected	HEP	Strategy 2: SSC^low^, CD123^pos^, HLA-DR^neg^	20	CD63	500	n.a.
Kvedariene et al., 2006	ROC, VEC, ATR, PANC, SUX	n.a	HEP	Strategy 1: SSC^low^, IgE^pos^	n.a.	CD63	1000	>15%
Monneret et al., 2002	ROC, VEC, ATR, MIV, SUX	Detected	n.a.	Strategy 3^++^: SSC^low^, CCR3^pos^, IgE^pos^, CD45^pos^	10	CD63	n.a.	>2%
Monneret et al., 2000	ROC, SUX, PROP, MDL, ALF, THI	Detected	n.a.	n.a.	n.a.	CD63	n.a.	n.a.
Sudheer and Appadurai, 2007	VEC, cATR, SUC	Not detected	HEP	n.a.	n.a.	CD63	n.a.	n.a.
Sudheer et al., 2005	ROC, VEC, ATR, ALC, SUX	Not detected	HEP	Strategy 1^+^: SSC^low^, IgE^pos^, CD45^pos^	15	CD63, CD203c	n.a.	>10%
Leysen et al., 2011	ROC, VEC	Detected	HEP	Strategy 2: SSC^low^, CD123^pos^, HLA-DR^neg^	n.a.	CD63	n.a.	>4%
**4. RADIO CONTRAST MEDIA (RCM)**								
Bohm et al., 2011	IOT, IOP	n.a	HEP	Strategy 1: SSC^low^, IgE^pos^	30	CD63	n.a	>5%
Pinnobphun et al., 2011	IOP, PM, IOB, IOH, IOX	n.a	EDTA	Strategy 3^+^: SSC^low^, CCR3^pos^, IgE^pos^	n.a	CD63	500	
Salas et al., 2013	IOH, IOD, IOM, IOB	Detected	n.a.	Strategy 1: SSC^low^, IgE^pos^	10^*^ + 20	CD63	1000	SI(%) > 2
Trcka et al., 2008	IOP, IOM, IPN	n.a	EDTA	Strategy 1: SSC^low^, IgE^pos^	n.a	CD63	500	>5%
**5. PLATIN CHEMOTHERAPEUTICS**								
Iwamoto et al., 2014	CPT	detected	EDTA	n.a	30	CD203c	n.a	>2%
Iwamoto et al., 2012	CPT	n.a	EDTA	Strategy 5: SSC^low^, CRTH-2^pos^, CD3^neg^	30	CD203c	n.a	>2%
**6. NON-STEROIDAL ANTI-INFLAMMATORY DRUGS (NSAIDs)**								
Harrer et al., 2010	DF	Not detected	EDTA	Strategy 3: SSC^low^, CCR3^pos^	20	CD63	500–1000	≥%, SI ≥ 2
Steiner et al., 2011	PP	Detected	EDTA	Strategy 3: SSC^low^, CCR3^pos^	45	CD63	500	≥5%
Abuaf et al., 2012	ASA, ACET, KET, DF, CEL	n.a	HEP	Strategy 4: SSC^low^, CD33^dim^, CD45^pos^	30	CD63, CD203c	400	Mean n.c. + 2 × *SD*
Bavbek et al., 2009	ASA, DF	n.a	ACD	Strategy 5: SSC^low^, CRTH-2^pos^, CD3^neg^	40, 15	CD63, CD203c	n.a.	≥5%, SI > 2
Celik et al., 2009	ASA	n.a	EDTA	Strategy 6: SSC^low^, FceRI^pos^	180	CD63, CD203c	500	MFI, SI > 2
Gamboa et al., 2004b	ASA, ACET, MET, DF, NAP	n.a	ACD	Strategy 1: SSC^low^, IgE^pos^	40	CD63	500	≥5%, SI ≥ 2
Gamboa et al., 2003a	MET	n.a	ACD	Strategy 1: SSC^low^, IgE^pos^	40	CD63	500	≥5%, SI ≥ 2
Gomez et al., 2009	MET	n.a	HEP	Strategy 1: SSC^low^, IgE^pos^	30	CD63	250-500	≥5%, SI > 2
Hagau et al., 2013	DIP	n.a	EDTA	Strategy 3: SSC^low^, CCR3^pos^	15	CD63	500	≥5%, SI ≥ 1.71
Kim and Cho, 2012	ASA, IBU, DF	n.a	EDTA	Strategy 1: SSC^low^, IgE^pos^	40	CD63	n.a.	≥5%, SI ≥ 2
Korosec et al., 2011	ASA	n.a	HEP	Strategy 2: SSC^low^, CD123^pos^, HLA-DR^neg^	15	CD63	500	% CD63 positive
Malbran et al., 2007	DF	n.a	HEP	Strategy 1^+^: SSC^low^, IgE^pos^, CD45^pos^	15	CD63	200	Mean n.c. + 2 × *SD*
Sanz et al., 2005	ASA, ACET, MET, DF, NAP	n.a	ACD	Strategy 1: SSC^low^, IgE^pos^	40	CD63	500	≥5%, SI ≥ 2
Phillips-Angles et al., 2016	ETO, MET	Clinically excluded	HEP	Strategy 2: SSC^low^, CD123^*pos*^, HLA-DR^neg^	15	CD63	500	≥5%, SI ≥ 2
**7. OTHER DRUGS**								
Soriano Gomis et al., 2012	GA	Detected	n.a.	Strategy 1: SSC^low^, IgE^pos^	40	CD63	500	≥5%, SI ≥ 2
Aranda et al., 2010	MPS	Detected	HEP	Strategy 1: SSC^low^, IgE^pos^	n.a	CD63	1000	SI ≥ 2
Ben Said et al., 2010a	MPS	n.a.	n.a.	n.a.	n.a	CD203c	n.a	>2%
Gamboa et al., 2003b	OPZ	n.a	ACD	n.a.	n.a	CD63	n.a	SI
Apostolou et al., 2006	GF	n.a	HEP	Strategy 1: SSC^low^, IgE^pos^	20	CD63	n.a	>6%

1. Beta-lactam antibiotics: amoxicillin (AX), ampicillin (AMP), benzylpenicillin (BP), benzylpenicilloyl-PLL (BPN-PLL), cefuroxim (CFX), cephalosporins (CEPH), diketopiperazine (DKP), minor determinant mixture (MDM), penicillin G (PCN G), penicillin V (PCN V), penicilloyl polylysine (PPL); 2. Fluoroquinolones: ciprofloxacin (CPX), flumequin (FLU), levofloxacin (LVX), lomefloxacin (LMX), moxifloxacin (MOX), norfloxacin (NFX), ofloxacin (OFX), pipemidic acid (PPA); 3. NMBAs: alcuronium (ALC), alfentanyl (ALF), atracurium (ATR), cisatracurium (cATR), midazolam (MDL), mivacurium (MIV), morphine (MSO4), pancuronium (PANC), pholcodine (PHO), propofol (PROP), rocuronium (ROC), succinylcholine (SUC), suxamethonium (SUX), thiopental (THI), vecuronium (VEC); 4. RCM: iobitrididol (IBT), iodixanol (IDX), iohexol (IOH), iomeprol (IOM), iopamidol (IPM), iopentol (IPN), iopromide (IOP), iotrolan (IOT), ioxithalamate (IOX); 5. Chemotherapeutics: carboplatin (CPT), cisplatin (CsPT); 6. NSAIDs: acetaminophen (ACET), acetyl salicylic acid (ASA), celecoxib (CEL), diclofenac (DF), dipyrone (DIP), etoricoxib (ETO), ibuprofen (IBU), ketoprofen (KET), metamizol (MET), naproxen (NAP), propyphenazon (PP); 7. Other drugs: gelofusine (GF), glatiramer acetate (GA), methyolprednisolone (MPS), omeprazol (OPZ); ethylenediaminetetraacetic acid (EDTA), heparin (HEP), acid citrate dextrose (ACD); n.a., not available; SSC, side scatter; MFI, mean fluorescence intensity; n.c., negative control; n, number; SD, standard deviation; SI, stimulation index of MFI activated compared to n.c.; SI(%), stimulation index of %CD63-/CD203c-positive compared to n.c.; +/++ indicate use of one/two additional identification marker(s) on top of the respective gating strategy;

*, pre-stimulation in incubation buffer.

In addition to anaphylactic degranulation another type of basophil activation termed piecemeal degranulation has been described (Dvorak, 2005). This alternative activation mechanism may also lead to altered surface expression of activation markers which can be assessed by BAT (Hausmann et al., 2009; MacGlashan, 2010). Consequently, BAT has been recognized as a promising tool for *in vitro* diagnosis of allergy or other hypersensitivity reactions including immediate adverse reactions to various drugs (Hoffmann et al., 2015).

## Technical issues of basophil activation testing

Usually BAT is performed from either heparinized, citrate- or EDTA-anticoagulated whole blood collected from allergic/hypersensitive donors (Table 1). When EDTA is used as anticoagulant Ca^++^ has to be supplemented to enable proper degranulation. For *in vitro* stimulation of basophils the samples are incubated with the allergen/drug or buffer only (negative control) for several minutes to hours at 37°C. As positive control, anti-IgE antibodies, anti-FcεRI antibodies, and formyl-methionine-leucine-phenylalanine (fMLF) are used. Latter represents an alternative degranulation/activation stimulus and is important to demonstrate basophil functionality in case of donors whose basophils fail to react *in vitro* to IgE-mediated pathway stimulation, so-called non-responders (Eberlein et al., 2010; MacGlashan, 2013).

Next, basophil identification and activation markers are stained with fluorescently labeled antibodies, subsequently erythrocytes are lysed. Depending on the protocol, staining can be performed during basophil stimulation in a single step. Upon flow cytometric acquisition of at least 200, in the optimal case 500–1000 basophils, activation marker expression is compared between buffer-treated samples and allergen-/drug-stimulated basophils. Different evaluation strategies are used. Some studies set the cut-off for spontaneously activated basophils arbitrarily at 5%, whereas others use stimulation indices of %CD63-/CD203c-positive cells, i.e., SI(%), or mean fluorescence intensities (MFI) of activation markers, i.e., SI, compared to negative control (Table 1). For interpretation of BAT area under the dose curve (AUC) measurements have recently been postulated. These enable a combined evaluation of basophil reactivity, i.e., the dose (range) at which maximal response occurs, and basophil sensitivity, i.e., the dose at which half of the maximal response occurs. As the AUC representation incorporates partial energy, which may arise at high allergen concentrations, and can be calculated even in cases where responses do not fit the typical shape of dose–response curves, it is particularly useful for monitoring the efficacy in allergen-specific immunotherapy (Ebo et al., 2004; Hausmann et al., 2009; Hoffmann et al., 2015).

## Basophil activation test with drugs—background considerations

Small molecular weight drugs constitute haptens which are not capable of FcεRI crosslinking themselves (hapten concept; Pichler et al., 2011). They require conjugation to carrier molecules (Figure 1A), usually abundant blood proteins, for eliciting an immune reaction in susceptible individuals. Moreover, reactive intermediates may be formed by drug metabolism (pro-hapten concept; Park et al., 1998; Naisbitt et al., 2000). Therefore, the use of drug metabolites and hapten-carrier conjugates has been promoted for the investigation of drug hypersensitivity reactions (Himly et al., 2003; Harrer et al., 2010; Steiner et al., 2011, 2014). Of note, in a case of propyphenazone (PP) hypersensitivity basophils reacted in BAT solely upon stimulation with the drug-carrier conjugate but not with pure PP (Steiner et al., 2014). Nevertheless, BAT is most frequently performed with solutions of plain drugs, a consequence of lacking knowledge in regard to relevant determinants, metabolic intermediates, their reactive functions, required linker length to the carrier molecule, and hapten orientation.

Alternative to the hapten and pro-hapten concepts in DHRs, the p-i concept has become well-accepted, however, it primarily accounts for T cell-mediated delayed-type immune reactions against drugs such as lidocaine, sulfamethoxazole, lamotrigine, carbamazepine, p-phenylendiamine, etc. or against metals like in nickel contact dermatitis (Pichler et al., 2006). DHRs of this kind are elicited on a different time-scale, not discussed here, and an involvement of basophils is unlikely. A comprehensive overview can be gained from reviews by Pichler et al. (Pichler, 2003; Pichler et al., 2015).

## BAT for the evaluation of immediate drug hypersensitivity to different drug classes

In the following paragraphs the suitability of basophil activation as a biomarker for evaluating immediate hypersensitivity reactions to different drug classes is discussed. The assay parameters used and activation patterns observed in the cited studies are summarized in Table 1.

### Basophil activation in antibiotics or quinolone hypersensitivity

Immediate DHRs against beta-lactam antibiotics such as penicillin, amoxicillin, and cephalosporin have been broadly investigated and are most likely IgE-mediated. Diagnosis of beta-lactam allergy first place is based on skin prick and intradermal tests. Sensitivity of skin tests, however, does not exceed 50–70%. The *in vitro* diagnostic method of quantifying beta-lactam-specific IgE antibodies (Mangodt et al., 2015) is an important complementary information. Clinically validated tests for drug-specific IgE, however, are difficult to develop, require complex coupling reactions for attaching the drug hapten onto a solid phase for antibody recognition, and are available only for a limited number of antibiotics. Instead, simple drug dilutions are used in BAT. It thus, appears a promising tool for *in vitro* diagnosis of beta-lactam allergy. Several groups investigated the applicability of BAT in the diagnostic management of beta-lactam allergy (Sanz et al., 2002; Gamboa et al., 2004a; Torres et al., 2004, 2010a, 2011; Abuaf et al., 2008; De Week et al., 2009; Eberlein et al., 2010; Garcia-Ortega and Marin, 2010). BAT performance, however, varied between groups with a median sensitivity of 50% (range 22–55%) and specificity ranging from 79 to 100%. Quintessence of these studies is that BAT was superior to immunoassaying for drug-specific IgE, but not to skin testing. Importantly, skin testing and BAT do not necessarily corroborate each other. Positive skin testing has been confirmed by BAT only in about 50–60% of patients (De Week et al., 2009; Torres et al., 2010a), whereas up to one third of skin test-negative patients have been identified by BAT (De Week et al., 2009). The tenor across studies thus was that the role of BAT currently is complementary, respectively supplementary, in the diagnosis of beta–lactam allergy.

Factors possibly contributing to the observed variance in sensitivity of BAT may involve patients selection criteria such as severity of reactions and time elapsed since the reaction (optimum: 1–6 months), regional preferences in drug prescriptions, and whether the drugs tested allow identification of cross-reactors. Methodological variations such as differential activation times (range 20–40 min) and different activation markers (CD63 and/or CD203c) additionally complicate comparability of results.

One key question is why should basophils degranulate in an IgE-dependent fashion *in vitro* upon stimulation with a dilution of monomeric small antibiotic haptens? One theory is that the beta-lactam ring confers instability to the compound facilitating the conjugation of the drug to abundant blood proteins such as albumin, transferrin and/or immunoglobulins (Torres et al., 2016). Once attached to a carrier protein the side chain of the drug, the thiazolidine ring, or the conjugation site itself are immunogenic in susceptible individuals and capable of both, eliciting an IgE response and crosslinking of surface-bound IgE.

Beyond diagnostic purposes one important application of BAT is determining an IgE-mediated pathomechanism when drug-specific IgE cannot be evidenced. Wortmannin, for instance, is a strong inhibitor of phosphatidylinositol 3-kinase (PI3K) and inhibits basophil activation in response to FcεRI crosslinking but not to stimulation with fMLF. In patients with selective allergy to the beta-lactamase inhibitor clavulanic acid, presence of drug-specific IgE was suspected but not detected. Using BAT an IgE-mediated pathomechanism was confirmed as BAT became negative upon stimulation with clavulanic acid in presence of wortmannin (Torres et al., 2010b).

BAT may be useful also in the diagnosis of immediate hypersensitivity reactions to quinolones. Diagnosis of quinolone allergy mainly relies on patient history and clinical manifestation. Skin tests are hampered by false positive reactions due to skin irritation rendering the positive predictive value of skin testing close to chance results. Drug-specific IgE have been reported, however, validated assays do not exist (Manfredi et al., 2004; Aranda et al., 2011; Mayorga et al., 2013). Aranda et al. showed both drug-specific IgE and dose-responsiveness in BAT for ciprofloxacin, moxifloxacin and levofloxacin, and inhibition of BAT positivity with wortmannin (Aranda et al., 2011). Moreover, they reported a higher sensitivity of BAT compared to IgE testing. Two out of seven studies evaluating BAT in quinolone hypersensitivity were negative with 0% sensitivity (Seitz et al., 2009; Lobera et al., 2010). The patient collectives of these two studies were quite small though as only 6, respectively, 4 patients were included. Contrary, Rouzaire et al. (2012) tested 34 patients and reported a very good negative predictive value of BAT as quinolones were successfully reintroduced in 15 of the 17 patients (50%) who tested negative in BAT. They emphasized the importance of negative BAT results in quinolone hypersensitivity as criteria for provocation test and thus the opportunity to possibly and safely reintroduce the drug (Rouzaire et al., 2012). Photodegradation could be one cause for false negative results. This was shown for moxifloxacin as positive results doubled when BAT was performed in the dark (Mayorga et al., 2013). Taken together, BAT appears helpful in the management of fluoroquinolone hypersensitivity.

### Basophil activation in neuromuscular blocking agent hypersensitivity

Anaphylactic episodes during general anesthesia have severe implications for the patient. Neuromuscular blocking agents (NMBAs) including rocuronium, vecuronium, atracurium, cisatracurium, and suxamethonium account for >60% of such cases with a high degree of cross-reactivity within this drug group and even further to opioid antitussives such as codeine or morphine, with the existence of drug-specific IgE demonstrated (Baldo and Fisher, 1983; Vervloet et al., 1983; Sainte-Laudy et al., 2006; Ebo et al., 2007; Leysen et al., 2013). However, it has been recognized that IgE accounts for immediate DHRs against NMBAs only in ~50% of cases. Therefore, a number of studies evaluated BAT protocols aiming at a more reliable before-hand screening tool (Monneret et al., 2000, 2002; Sudheer et al., 2005; Ebo et al., 2006; Kvedariene et al., 2006; Sudheer and Appadurai, 2007). Sudheer et al. (2005) compared CD63, CD203c, histamine release and skin testing for their predictive values. While specificities reached 100%, the sensitivities of these four techniques determined for the whole NMBA group in their cohort of 21 patients resulted in 79, 36, 36, and 64%, respectively. Other studies reported specificities for BAT based on CD63 expression of >93% with sensitivities of >54%. As CD63 enables a better judgment of the type of basophil activation than CD203c, i.e., creating a log-shift in the flow cytometric dot plots, Ebo et al. (2006) were able to reach a sensitivity for DHR against rocuronium >91.7% using 0.5 mg/ml of drug and setting the diagnostic threshold value at 4% to be most discriminative, as they determined from two-graph receiver operating characteristics. In a large study involving thorough diagnostic workup of 104 patients the same group (Leysen et al., 2011) obtained a positive predictive value of 98% for rocuronium using the combination of skin testing, BAT, and drug-specific IgE testing by ImmunoCAP. In their study, skin tests turned out most reliable, however, when these are negative, BAT is indicated. For assessment of cross-reactivity between rocuronium and vecuronium, the two most often used NMBAs, BAT was found to complement skin tests well.

### Basophil activation in radiocontrast media hypersensitivity

Immediate DHRs against non-ionic radiocontrast media (RCM) occur in 0.7–3.1% of patients, with ionic RCM being even more prevalent, i.e., up to 12.7% including mild reactions or reactions due to rapid intravenous infusion of these highly osmolar substances (Stellato et al., 1996). Nevertheless, 0.04% of patients experience severe reactions upon administration of non-ionic RCM (Wolf et al., 1989; Katayama et al., 1990; Lieberman and Seigle, 1999). Traditionally, RCM reactions have been considered as non-IgE-mediated, and the majority of DHRs indeed seem to result from RCM non-specifically binding to surface receptors or indirectly interfering with the complement or kinin cascades, but skin testing and BAT have been successfully applied more recently (Brockow et al., 2009; Javaloyes et al., 2012; Philipse et al., 2013). Still few anecdotal reports on RCM-specific IgE exist, therefore, some studies have sought to differentiate IgE- from non-IgE-mediated mechanisms, however, have not determined the presence of drug-specific IgE themselves. Moreover, a number of studies have evaluated BAT as a diagnostic tool for RCM hypersensitivity, however, giving variable experimental details, which makes it difficult to judge the mode of basophil activation (Trcka et al., 2008; Bohm et al., 2011; Pinnobphun et al., 2011; Salas et al., 2013). Presence of drug-specific IgE was not shown in any of the reports. Some studies demonstrated log-shift activation of CD63 (Pinnobphun et al., 2011), while others did not (Bohm et al., 2011). Meta-analyzing the presented flow cytometric data some degree of inconsistency in the basophil activation profiles seems to be a common characteristic of immediate DHRs against RCM. Other influences may account as listed in a commentary by Chirumbolo (2013). For instance, a dose-dependent enhancement of CD63 expression upon pre-stimulation with interleukin 1β was observed by Boehm et al., however, the authors also reported variation of this effect between different RCM. Summarizing, one can say literature has demonstrated sensitivities of 46–63% and specificities of 89–100% for BAT qualified on the combination of skin and provocation testing serving as the current diagnostic standard for RCM hypersensitivity. In general, good correlation between skin test, drug provocation test, and BAT has proven useful to complement *in vivo* testing (Pinnobphun et al., 2011; Salas et al., 2013). Nevertheless, only in rare cases drug-specific IgE is involved.

### Basophil activation in platinum-containing chemotherapeutic hypersensitivity

The DHRs against carboplatin or cisplatin, compounds used repeatedly at high doses in anti-cancer therapy, include hyper- or hypotension, vomiting, dyspnea, and wheezing. They arise in up to 26.7% of patients and skin testing has been successfully performed although safety concerns exist regarding severe side-effects for the patient and risk of exposure of the medical personnel (Markman et al., 1999; Leguy-Seguin et al., 2007; Sugimoto et al., 2011). Consequently, the potential of BAT for predicting DHRs has been investigated using flow cytometric determination of CD203c (Iwamoto et al., 2012, 2014). These authors verified the involvement of IgE in carboplatin-induced DHR, as the basophil activation could be inhibited by wortmannin and anti-IgE pretreatment by omalizumab. In a case study of a severely anaphylactic history, BAT was applied evaluating the potential additive histamine-liberating effect by cisplatin following RCM administration (Viardot-Helmer et al., 2008). In conclusion, a similar situation to RCM seems to occur with platinum-containing anti-neoplastic drugs. In a low number of patients drug-specific IgE seems to be involved, still BAT may be suitable for diagnosis of severe immediate DHRs even if pharmacologic, i.e., non-immune-mediated mechanisms are underlying.

### Basophil activation in analgesic hypersensitivity

Non-steroidal anti-inflammatory drugs (NSAIDs) are known to frequently cause drug hypersensitivity reactions ranging from mild symptoms, e.g., skin reactions, to severe life-threatening systemic complications (Kowalski et al., 2011). Although the symptoms can mimic typical IgE-mediated reactions, drug-specific IgE seems not to be involved in most NSAID hypersensitivities, as NSAID-specific IgE has only been detected for PP (Himly et al., 2003). Investigating severe selective diclofenac (DF) hypersensitivity using drug-human serum albumin conjugates of DF and the most common DF metabolites no drug-specific IgE was detectable by ELISA (Harrer et al., 2010). Instead of an IgE-mediated mechanism underlying immediate DHRs to DF, it has been hypothesized that the inhibition of cyclooxygenase-1, triggering the reduction of prostaglandin E2 accompanied by an increase in leukotriene production, accounts for the observed type I-like symptomatic (Mastalerz et al., 2004). Regardless the lack of NSAID-specific IgE, BAT has been explored by several groups leading to conflicting results (Gamboa et al., 2003a, 2004b; Celik et al., 2009; Gomez et al., 2009; Sanz et al., 2005; Malbran et al., 2007; Bavbek et al., 2009; Harrer et al., 2010; Korosec et al., 2011; Steiner et al., 2011; Abuaf et al., 2012; Kim and Cho, 2012; Hagau et al., 2013; Phillips-Angles et al., 2016). Sensitivities varied from 0 (Harrer et al., 2010; Steiner et al., 2011) to 80% (Korosec et al., 2011) and specificities from 40 (Celik et al., 2009) to 100% (Gamboa et al., 2003a, 2004b; Hagau et al., 2013). Reasons may be manifold as single NSAIDs and different combinations of NSAIDs were tested and activation times ranged from 15 min to 3 h. In contrast to the negative BAT results, a most recent case report showed that basophils of an etoricoxib-hypersensitive patient were tested positive upon stimulation with the parent drug, although the authors excluded an involvement of drug specific IgE (Phillips-Angles et al., 2016). Here and in most other studies CD63 was the most commonly used activation marker. Introduction of CD203c by some groups (Bavbek et al., 2009; Celik et al., 2009; Abuaf et al., 2012) did not make results less ambiguous. Korosec et al. (2011) pointed out that a considerate pre-selection of most severe cases of DHRs may lead to an improvement in sensitivity and specificity of BAT. Another important factor for investigating NSAID hypersensitivity may be the time interval between the incident and BAT which should be less than 18 months according to the EAACI position paper on BAT (Hoffmann et al., 2015) or less than 6 months according to Gomez et al. (2009). To put it all in a nutshell, due to the many contradictory data regarding sensitivity and specificity, potentially resulting from differences in gating protocols influencing basophil activation (Chirumbolo, 2014), further large-scale trials following harmonized BAT protocols including data interpretation are needed to determine suitability of BAT for diagnosis of NSAID hypersensitivity.

### Basophil activation in hypersensitivity to other drugs and some remarks on biologicals

Various other drugs causing immediate DHRs have been tested using BAT including atropine (Cabrera-Freitag et al., 2009), glatiramer acetate (Soriano Gomis et al., 2012), methylprednisolone (Aranda et al., 2010; Ben Said et al., 2010a), omeprazole (Gamboa et al., 2003b), the diuretic hydrochlorothiazide (Gamboa et al., 2005; Manso et al., 2010), or antihistamines (Caceres Calle and Fernandez-Benitez, 2004; Bobadilla-Gonzalez et al., 2011; Lee et al., 2011; Sanchez Morillas et al., 2011) to name a few. However, these reports, most of them case studies, are hard to judge from the perspective of validating BAT performance, as no experimental details were presented. Aranda et al. (2010) showed log-shifts in CD63 expression in the dot plots and indicated involvement of methylprednisolone-specific IgE as activation could be inhibited by wortmannin. Accordingly, Soriano Gomis et al. (2012) determined drug-specific IgE in their workup, however, no details on how BAT was conducted were given. Unequivocal BAT performance with 100% sensitivity and 87.5% specificity evidenced by log-shifts in CD63 expression shown in dot plots was reported for Gelofusine® hypersensitivity of 6 patients (Apostolou et al., 2006).

Adverse reactions to biologicals are increasing due to their expanding utilization, as reviewed recently (Corominas et al., 2014; Galvao and Castells, 2015). For instance, rituximab hypersensitivity with an incidence of 5–10% has been evaluated using BAT in a cohort of 18 B cell lymphoma patients (Piva et al., 2012). The authors reported successful discrimination between patients and controls based on CD63 expression at *in vivo* concentrations, however, no experimental details were presented. Very recently an interesting new approach to predict DHRs against cetuximab during cancer immunotherapy based on evaluating the decrease in cetuximab molecules on basophils after dissociation of IgE from FcεRI was reported (Iwamoto et al., 2016).

Notably, Werner J. Pichler has proposed a new classification for biologicals in five classes (α-ε), as biologicals differ in that they represent intact antigens themselves and are not metabolized like small molecular weight drugs (Pichler, 2006). We will thus not discuss ADRs against biologicals here.

## Conclusions

Immediate DHRs present a complex phenomenon in regard to etiology. Similar clinical phenotypes are observed in “type I DHRs” with—according to the (pro-)hapten concept—drug-specific IgE against e.g., antibiotics, half of NMBAs, PP, or biologicals, and in “type I-like DHRs” elicited by alternative activation pathways as it is most likely the case with the other half of NMBAs, most RCM, platinum-containing chemotherapeutics, or NSAIDs. A close look on the CD63 upregulation pattern may help differentiating, as in type I DHRs the sudden basophil degranulation usually results in a log-shift of signal intensity. In contrast, no unequivocal conclusions on the underlying mechanism may be drawn when a “smear-like” CD63 upregulation is observed. In such cases, especially if basophil activation is weak, type I-like mechanisms independent of IgE cannot be excluded but bear the risk of false positive interpretation. We, therefore, recommend inclusion of plots/histograms in the publications to allow the reader evaluating the basophil activation profile. To circumvent bystander effects caused by interaction with other cells basophil purification protocols, as described recently (Steiner et al., 2016), may be considered, in particular when investigating more specific questions beyond diagnosis. Overall, the basophil response in BAT is influenced by many so far unpredictable factors which may impair the quality of results in certain cases (Chirumbolo, 2016).

Finally, we shall not forget that basophil activation test is an *in vitro* surrogate marker for a systemic reaction of the entire organism. This renders skin and/or provocation testing still of primary importance for diagnosis. Nevertheless, BAT has qualified a safe and suitable *in vitro* complement of *in vivo* testing in immediate DHRs, as most recently pointed out by a position paper of the European Network for Drug Allergy (ENDA) and the EAACI Drug Allergy Interest Group (Mayorga et al., 2016).

## Author contributions

MS, AH, and MH were involved in the concept, literature screening, and writing of the article.

### Conflict of interest statement

The authors declare that the research was conducted in the absence of any commercial or financial relationships that could be construed as a potential conflict of interest.
